# Phosphocholine-Specific Antibodies Improve T-Dependent Antibody Responses against OVA Encapsulated into Phosphatidylcholine-Containing Liposomes

**DOI:** 10.3389/fimmu.2016.00374

**Published:** 2016-09-22

**Authors:** Yoelys Cruz-Leal, Alejandro López-Requena, Isbel Lopetegui-González, Yoan Machado, Carlos Alvarez, Rolando Pérez, María E. Lanio

**Affiliations:** ^1^Laboratory of Toxins and Liposomes, Center for Protein Studies (CEP), Faculty of Biology, University of Havana, Havana, Cuba; ^2^Immunobiology Direction, Center of Molecular Immunology (CIM), Havana, Cuba; ^3^Biochemistry Department, Faculty of Biology, University of Havana, Havana, Cuba; ^4^Development Direction, Center of Molecular Immunology (CIM), Havana, Cuba

**Keywords:** B-1 cells, liposomes, phosphocholine-specific antibodies, peritoneal macrophages, humoral response

## Abstract

Liposomes containing phosphatidylcholine have been widely used as adjuvants. Recently, we demonstrated that B-1 cells produce dipalmitoyl-phosphatidylcholine (DPPC)-specific IgM upon immunization of BALB/c mice with DPPC-liposomes encapsulating ovalbumin (OVA). Although this preparation enhanced the OVA-specific humoral response, the contribution of anti-DPPC antibodies to this effect was unclear. Here, we demonstrate that these antibodies are secreted by B-1 cells independently of the presence of OVA in the formulation. We also confirm that these antibodies are specific for phosphocholine. The anti-OVA humoral response was partially restored in B-1 cells-deficient BALB/*xid* mice by immunization with the liposomes opsonized with the serum total immunoglobulin (Ig) fraction containing anti-phosphocholine antibodies, generated in wild-type animals. This result could be related to the increased phagocytosis by peritoneal macrophages of the particles opsonized with the serum total Ig or IgM fractions, both containing anti-phosphocholine antibodies. In conclusion, in the present work, it has been demonstrated that phosphocholine-specific antibodies improve T-dependent antibody responses against OVA carried by DPPC-liposomes.

## Introduction

IgM is the first antibody isotype to appear during ontogeny and the only isotype produced by all species of vertebrates (1, 2). It is also the first isotype produced during an immune response and plays a crucial role in front-line host defense against pathogens. Secreted IgM plays important roles in the early phases of the adaptive immune response, as it concentrates antigen into secondary lymphoid organs, initiates antibody responses and germinal center formation, and accelerates affinity maturation in immune responses to thymus-dependent antigens (1, 3, 4).

Up to 80% of circulating IgM in the mouse derives from B-1 cells (5). B-1 cells represent the main B cell population of the peritoneal and pleural cavities in mice (6) and differ from conventional B lymphocytes (B-2) in surface markers, antibody repertoire, developmental pathway, and B-cell receptor (BCR) signaling (7). CD5 expression splits B-1 cells into two subsets: CD5^+^ B-1a and CD5^−^ B-1b cells, which exhibit different functions in the immune system (8, 9). The natural repertoire of peritoneal B-1 cells contains phosphocholine and phosphatidylcholine-specific antibodies, and hence they might interact with liposomes composed by this lipid (10–12). The so-called natural antibodies, mainly produced by these cells, are present in circulation without any evident antigenic challenge (13). The most studied natural antibodies bind to phosphorylcholine-containing antigens, which are present and accessible on apoptotic cell membranes and in oxidized low density lipoproteins, and also constitutes the immunodominant epitope in the pneumococcal cell wall polysaccharide (CW-PSC) (14–16). There is a distinct set of natural antibodies that bind to determinants that arise on erythrocytes during their senescence or after enzymatic treatment with bromelain (10, 17–19). These anti-red cell antibodies are reported to recognize determinants that involve the entire phosphatidylcholine molecule in the outer cell membrane, but not other phosphocholine-containing antigens.

Recently, we demonstrated the contribution of B-1 cells to the adjuvant properties of dipalmitoyl-phosphatidylcholine (DPPC) and cholesterol (Chol)-containing liposomes (Lp DPPC) encapsulating ovalbumin (OVA) (Lp DPPC/OVA) (20). BALB/X-linked immunodeficient (*xid*) mice, which exhibit defects in the B cell compartment, particularly in the B-1 cell population (21–23), showed quantitative and qualitative differences in the anti-OVA antibody response compared with wild-type animals upon immunization with this preparation. The direct participation of B-1 cells was evidenced by the restoration of the immunostimulatory properties of Lp DPPC in BALB/*xid* mice adoptively transferred with B-1 cells purified from BALB/c animals; the internalization of these particles by B-1 cells; and the migration of B-1 cells from the peritoneal cavity (PerC) to the spleen. These cells were able to produce both *in vitro* and *in vivo* DPPC-specific antibodies upon stimulation with Lp DPPC (20). These antibodies recognized sphingomyelin (SM) but not dipalmitoyl-phosphatidylglycerol (DPPG), suggesting their phosphocholine specificity. However, the precise contribution of these antibodies to the enhancement of the OVA-specific antibody response promoted by Lp DPPC encapsulating this antigen was not elucidated. In the present work, we characterized the anti-lipid antibody response induced by this liposomal preparation, its specificity, and the influence of the presence of the antigen. The presence of OVA in the formulation did not increase the anti-DPPC IgM response. These antibodies also recognized the CW-PSC from *Streptococcus pneumoniae*, corroborating their specificity for phosphocholine. The opsonization of Lp DPPC/OVA with these antibodies enhanced the anti-OVA humoral response in B-1 cells-deficient BALB/*xid* mice, although without reaching the levels obtained in wild-type animals. The particles opsonized with serum total immunoglobulin (Ig)- or IgM-containing phosphocholine-specific antibodies were efficiently phagocyted by peritoneal macrophages, suggesting a role for these cells in the adjuvant properties of Lp DPPC.

## Materials and Methods

### Reagents

OVA grade V, used as model antigen in immunization protocols in soluble form or encapsulated into liposomes and OVA grade II, used to coat ELISA plates, were purchased from Sigma–Aldrich (St. Louis, MO, USA). CW-PSC from *S. pneumoniae* used to coat ELISA plates was purchased from Statens Seruminstitut (Copenhagen, Denmark). DPPC, DPPG, and Chol, used to generate liposomes and to coat ELISA plates, were purchased from Northern Lipids (Alabaster, AL, USA). Dimyristoyl-phosphatidylcholine (DMPC), distearoyl-phosphatidylcholine (DSPC), dioleoyl-phosphatidylcholine (DOPC), SM, phosphatidic acid (PA), phosphatidylserine (PS), and phosphatidylethanolamine (PEt), used to coat ELISA plates, were purchased from Avantis Polar Lipids, Inc., Alabaster, AL, USA. Fluorescein isothiocyanate (FITC) from Sigma–Aldrich was used to label OVA. Dephosphorylated 18C polysaccharide from *S. pneumoniae* (dephos 18C PSC), used in the competitive ELISA, was generously provided by Dr. Janoi Chang from the Finlay Institute, Havana, Cuba.

### Mice

Female BALB/c mice, 6 to 8 weeks of age, were purchased from the Center for Laboratory Animal Production (Havana, Cuba). Female and male BALB/*xid* mice, which carry a Bruton’s tyrosine kinase mutation and have a severely diminished B-1 cell population (13, 23), were bred at the Center of Molecular Immunology (CIM; Havana, Cuba). All animals were specific pathogens-free and were maintained under standard animal house conditions with free access to water and standard rodent pellets.

### Ethics Statement

All procedures were performed in compliance with the protocols approved by the Institutional Committee for the Care and Use of Laboratory Animals of the CIM (CICUAL, 0017/2008). Animals were sacrificed by cervical dislocation, minimizing their suffering.

### Encapsulation of OVA into Liposomes

Liposomes encapsulating OVA were obtained by a procedure based on dehydration and rehydration of vesicles (DRV) developed by Kirby and Gregoriadis (24). To obtain OVA-encapsulating liposomes, small unilamellar vesicles (SUV) composed of DPPC and an equimolar quantity of Chol were generated by ultrasonication and then mixed with OVA. After freezing at −70°C, the liposome and OVA mixture was lyophilized in an Edwards freezer dryer (Aaron Equipment Company, Bensenville, IL, USA) for 24 h. The rehydration step was carried out with a small volume of distilled water (1 μL water/0.2 μmol of lipids) at 45°C, above the phase transition temperature of DPPC. After incubating for 30 min at 45°C, 0.5 mL of phosphate-buffered saline (PBS), pH 7.4, was added. Separation of non-encapsulated OVA was performed by centrifugation at 100,000 *g* for 30 min (Centrifuge 5415 R, Eppendorf AG, Hamburg, Germany). Empty liposomes comprised of DPPG and Chol (Lp DPPG), DPPC and Chol (Lp DPPC), or DPPG, PA, and Chol in a ratio 0.25:0.75:1 (Lp DPPG:PA:Chol) (Lp DPPG:PA) were prepared following the same procedure, but in the absence of OVA.

### Binding of Antibodies Induced by DPPC-Containing Liposomes to CW-PSC

The recognition of CW-PSC by antibodies induced by Lp DPPC was tested by ELISA. 96-well polystyrene flat-bottom high binding microtiter plates (Maxisorp; Nunc, Roskilde, Denmark) were coated with 10 μg mL^−1^ of CW-PSC from *S. pneumoniae* diluted in PBS, pH 7.2, overnight at 4°C. The plates were blocked with 5% (w/v) skim milk (Merck, Darmstadt, Germany) in PBS/0.05% Tween 20 (PBS/T) (v/v) (block solution I) for 1 h at 37°C. Serial dilutions of preimmune and immune serum samples were incubated overnight at 4°C. Bound antibodies were detected with a biotinylated goat anti-mouse IgM antibody (AbD Serotec, Oxford, UK) followed by alkaline phosphatase-conjugated streptavidin (Sigma–Aldrich). A serum from a human donor immunized with the 7-valent pneumococcal polysaccharide–protein conjugate vaccine (PCV7; Prevnar^®^, Wyeth Lederle Vaccines) was used as positive control of CW-PSC recognition, and binding detected with a biotinylated goat anti-human IgM antibody (Jackson ImmunoResearch Laboratories Inc., West Grove, PA, USA) followed by alkaline phosphatase-conjugated streptavidin (Sigma–Aldrich). Chromogen *p*-nitrophenylphosphate diluted in diethanolamine/MgCl_2_ buffer, pH 5, was added as substrate solution and optical density read at 405 nm (OD_405 nm_) in a plate reader (ELISA Ledial01, Wiener Neudorf, Austria). Wells without coating and wells coated with CW-PSC and incubated only with the secondary antibodies were used as background controls.

For the competitive ELISA, plates coated with 10 μg mL^−1^ of CW-PSC from *S. pneumoniae* were incubated with immune sera from mice immunized with Lp DPPC previously mixed with different concentrations of Lp DPPC as competitor molecule. CW-PSC was used as positive control and Lp DPPG:PA and dephos 18C PSC as negative controls. Solutions of PSC at 50 and 12 μg mL^−1^ and the liposomes at 80 and 20 μg mL^−1^ were mixed with the immune mouse sera diluted 1:100 in a ratio 1:1 (v/v) and incubated for 2 h at 37°C. Sera with or without competitor molecule were added to the plate and incubated overnight at 4°C. After three washes, bound antibodies were detected with a biotinylated goat anti-mouse IgM antibody (AbD Serotec) followed by alkaline phosphatase-conjugated streptavidin (Sigma–Aldrich). The reaction was developed as describe above. The percentage of binding of immune sera in the presence of competitor molecule was determined with respect to signal in the absence of competitor molecule.

### IgM Purification from Sera of Mice Immunized with Empty Liposomes

To purify IgM fractions from sera of BALB/c mice immunized with empty Lp DPPC or Lp DPPG, Ig fractions were precipitated with NH_4_SO_4_ and applied into a column of agarose with covalently attached goat anti-mouse IgM (μ-chain-specific) IgG fraction (Sigma–Aldrich). After washing the column with 0.01M sodium phosphate buffer, pH 7.2, containing 0.5M NaCl (PB), the elution step was carried out with 0.1M glycine with 0.15M NaCl, pH 2.4. Finally, the IgG contaminant was eliminated using a Hi-Trap protein G column (GE Healthcare Bio-Sciences AB, Uppsala, Sweden). The unbound fraction (IgM) was collected by washing the column with PB and the bound fraction (IgG) by eluting with 0.1M glycine–HCl buffer, pH 2.7. Both chromatographic steps were performed at a flow rate of 1 mL min^−1^. Eluted fractions were neutralized using 200 μL of 1M Tris–HCl, pH 9.0. The protein concentration was estimated by absorbance at 280 nm. Polyacrylamide gel electrophoresis (SDS-PAGE) (25) and Western blotting analysis were performed to assess the purity of samples, using alkaline phosphatase-conjugated goat anti-mouse IgM (μ-chain-specific) and anti-mouse IgG (whole molecule) antibodies (Jackson ImmunoResearch), respectively. Molecular weight markers (Precision Plus Protein™ All Blue Standards) and IgM/IgG standards were purchased from Bio-RAD (Waltham, MA, USA) and Sigma–Aldrich, respectively. In addition, IgM and IgG concentration was estimated by ELISA. The specificity of the IgM fraction for DPPC was also checked by ELISA.

### IgM and IgG Quantification by ELISA

To quantify the IgM fraction, 96-well polystyrene flat-bottom high binding microtiter plates (Corning™ Costar™, Thermo Fisher, Toronto, ON, Canada) were coated with a goat anti-mouse IgM (μ-chain-specific) antibody (Sigma–Aldrich), diluted 1:3500 in 0.05M sodium carbonate buffer, pH 9.6 (coating buffer). The plates were incubated overnight at 4°C and blocked with 1% (w/v) of bovine serum albumin diluted in PBS (block solution II) for 30 min at 37°C. The samples diluted in block solution II were added and incubated for 2 h at 4°C. Serial dilutions (1:2) of an irrelevant mouse IgM (Sigma–Aldrich) were used as standard curve. Bound antibodies were detected with an alkaline phosphatase-conjugated goat anti-mouse IgM (μ chain-specific) antibody (Jackson ImmunoResearch), diluted 1:10,000 in block solution II after incubation for 1 h at 4°C. The reaction was developed as described above.

The IgG fraction was quantified using a similar assay, with a goat anti-mouse IgG (whole molecule) antibody (Sigma–Aldrich) diluted 1:333 as capture antibody, and samples diluted in PBS-Tween 20 (0.05%) with fetal bovine serum (5%) (PBS-FBS). Serial dilutions (1:2) of an irrelevant mouse IgG, produced at CIM (Havana, Cuba) were used as standard curve. After 1 h of incubation at 37°C, bound antibodies were detected using an alkaline phosphatase-conjugated goat anti-mouse IgG (Fcγ fragment-specific) (Jackson ImmunoResearch) diluted 1:10,000 in PBS-FBS.

### Liposome Opsonization by Phosphocholine-Specific Antibodies

The opsonization assay was carried out by incubating Lp DPPC/OVA with serum total Ig and IgM fractions for 2 h at 37°C. Bound antibodies were detected by flow cytometry using a PE-conjugated goat anti-mouse Ig antibody and PE-conjugated goat anti-mouse IgM, respectively (eBioscience, San Diego, CA, USA). To opsonize the amount of Lp DPPC/OVA corresponding to one immunization dose, molar ratios antibodies:lipids of 0.01:0.792 and 0.0001:0.792 were used for serum total Ig and IgM fractions, respectively.

### Immunization Protocols

The schedule followed in all intraperitoneal (i.p.) immunization protocols was one injection at day 0 and a booster after 14 days. Animals were bled at day 0 and 7 days after the booster. Anti-OVA and anti-lipid antibody responses were evaluated by ELISA. BALB/*xid* mice were also immunized with OVA labeled with FITC (FITC-OVA) encapsulated into Lp DPPC (Lp DPPC/FITC-OVA) opsonized or not with anti-phosphocholine antibodies-containing serum total Ig fraction.

### Determination of Serum Antibodies Specific for OVA and Lipids

To detect OVA-specific antibodies, 96-well polystyrene flat-bottom high binding microtiter plates (Greiner-bio-one, Frickenhausen, Germany) were coated with 10 μg mL^−1^ of OVA diluted in coating buffer, overnight at 4°C. The plates were blocked with block solution I for 1 h at 37°C. Serial dilutions of serum samples were incubated for 2 h at 37°C. Bound antibodies were detected with an alkaline phosphatase-conjugated goat anti-mouse IgG antibody (Sigma–Aldrich) or biotinylated goat anti-mouse IgG1 or IgG2a antibodies (AbD Serotec) followed by alkaline phosphatase-conjugated streptavidin (Sigma–Aldrich). Serum dilutions giving signals corresponding to twice the value with the preimmune sera were considered as antibody titers.

To evaluate the presence of phosphocholine-specific antibodies, 96-well polystyrene flat-bottom microtiter plates (Maxisorp; Nunc) were coated with 4 μg of DPPC, DSPC, DOPC, DMPC, DPPG, SM, PA, PS, or PEt diluted in n-hexane and incubated at 37°C until drying. The plates were blocked with 5% (w/v) skim milk (Merck) in PBS (blocking solution III) for 1 h at 37°C. Serum samples were diluted in blocking solution III, and plates were incubated overnight at 4°C. Bound antibodies were detected with a biotinylated goat anti-mouse IgM antibody (AbD Serotec) or a biotinylated goat anti-mouse IgG antibody (Sigma–Aldrich) followed by alkaline phosphatase-conjugated streptavidin (Sigma–Aldrich).

In both anti-OVA and anti-lipid determinations, the reaction was developed as described above.

### Evaluation of Opsonized Lp DPPC/OVA Uptake by Peritoneal Macrophages *In Vivo*

BALB/*xid* mice (*n* = 3) were immunized i.p. with Lp DPPC/FITC-OVA opsonized or not with anti-phosphocholine antibodies-containing serum total Ig fraction (Lp DPPC/FITC-OVA + Ab and Lp DPPC/FITC-OVA, respectively). The IgM fraction from sera of BALB/c mice immunized with empty Lp DPPC (IgM_DPPC_) or Lp DPPG (IgM_DPPG_) and a commercial irrelevant IgM (IgM_irrelev_) (Sigma–Aldrich) were also used to opsonize Lp DPPC/FITC-OVA. One hour later, cells were collected from the PerC by repeated washing with RPMI 1640 medium (Sigma–Aldrich) and labeled with the following goat anti-mouse antibodies combinations: PE-conjugated anti-F4/80 (PE-F4/80); cyanine dye (Cy5.5) combined with PE-conjugated anti-CD11b (PE Cy5.5-CD11b) and eFluor700-conjugated anti-CD19 (eFluor700-CD19) or PE-conjugated anti-CD11b (PE-CD11b); PE cy-chrome 5 (Cy5)-conjugated anti-F4/80 (PE Cy5-F4/80), and allophycocyanin-conjugated anti-B220 (APC-B220). Macrophage populations were identified by flow cytometry from total cells as CD19^−^CD11b^+^ and F4/80^+^ (F4/80^Low^ and F4/80^High^) (Figure S1 in Supplementary Material). Cells from non-immunized BALB/*xid* mice were used as negative control.

### Flow Cytometry Analysis

For phenotype characterization, cell suspensions were preincubated with an anti-CD16/CD32 mAb (BD Biosciences Pharmingen, San Diego, CA, USA) to block Fcγ II/III receptors before staining with fluorochrome-conjugated antibodies. Cells were stained with different combinations of goat anti-mouse antibodies: PE-F4/80, PE-CD11b, PE-Cy5-F4/80, PE Cy5.5-CD11b, APC-B220, and eFluor700-CD19 using standard protocols. Cells were acquired using a Gallios flow cytometer (Beckman Coulter, Miami, FL, USA). The analysis was performed using the FlowJo 7.2.2 software (Tree Star Ashland, OR, USA). Total number of macrophages was estimated by total cell number in the PerC counted in a Neubauer chamber.

### Statistical Analysis

Statistical analysis was performed using the SPSS software version 16.0 (SPSS). The Kolmogorov–Smirnov test was used to verify normal distribution of data and the Levene test to determine the homogeneity of variance. Data with normal distribution and equality of variance were analyzed with one-way variance analysis (ANOVA) simple classification, with Tukey as *post hoc* test to assess statistical significance between the means of more than two groups. Data not normally distributed or without equality of variance, even after scale transformation, were analyzed using the Kruskal–Wallis non-parametric test with Dunn as *post hoc* test and the Friedman test with Dunn as *post hoc* test for matched data. For comparing the means of two independent groups, the Mann–Whitney *U* test or the Wilcoxon signed-rank test were used.

## Results

### B-1 Cells Produce Anti-Liposomal DPPC Antibodies with Specificity for Phosphocholine

**Figure 1:**
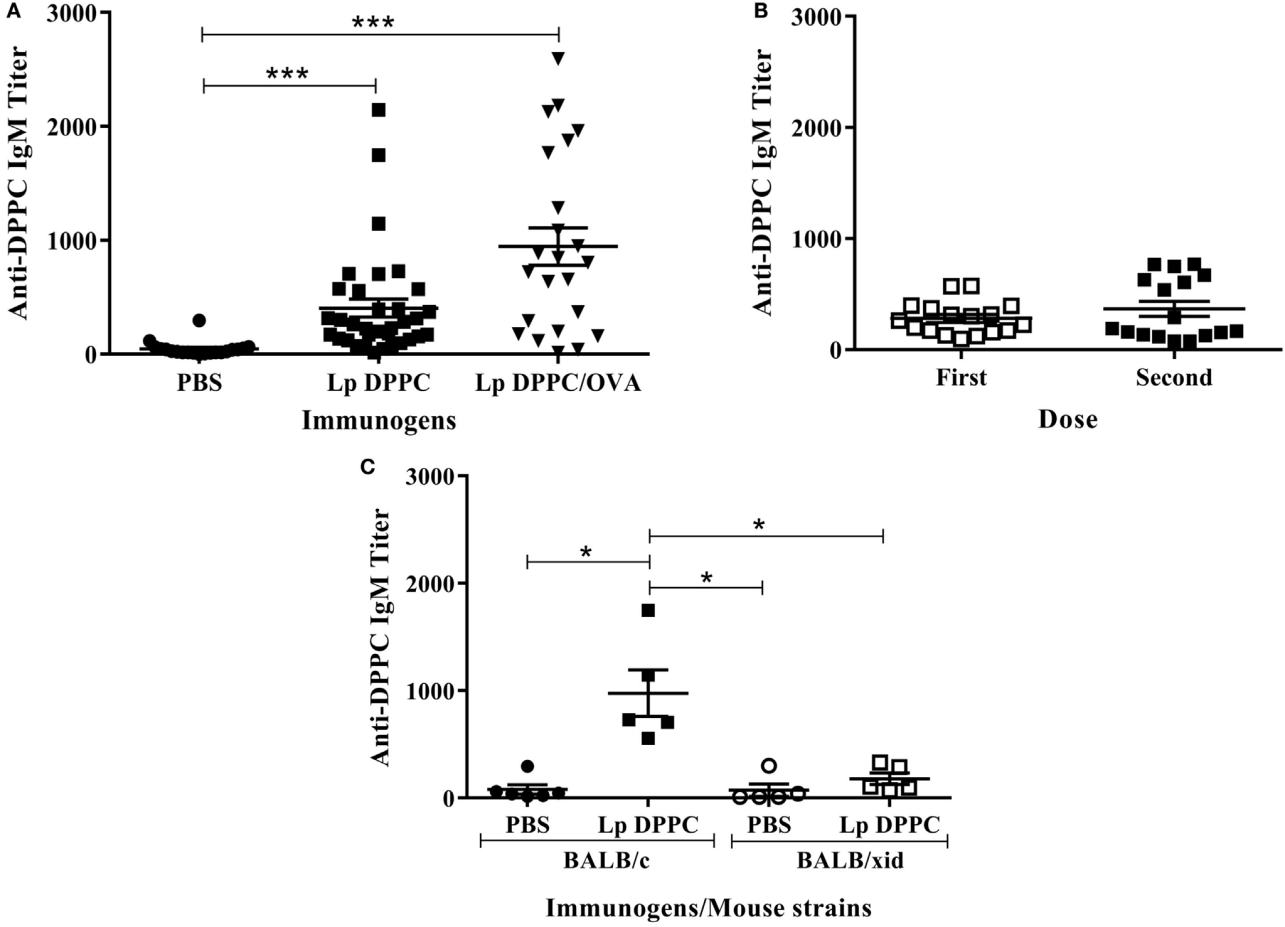
**Liposomal DPPC induces DPPC-specific IgM in BALB/c, but not in BALB/*xid* mice**. DPPC-specific IgM titers were evaluated by ELISA in **(A)** sera from BALB/c mice immunized i.p. with two doses of PBS (*n* = 20), Lp DPPC (*n* = 28), or Lp DPPC/OVA (*n* = 23); **(B)** sera from BALB/c mice (*n* = 17) immunized i.p. with one or two doses of Lp DPPC; **(C)** sera from BALB/c and BALB/*xid* mice immunized i.p. with two doses of PBS or Lp DPPC (*n* = 5 in each group). Means ± SEM are shown. Statistical analysis was performed with Kruskal–Wallis test with Dunn posttest **(A,C)** and Wilcoxon signed-rank test **(B)**; *p < 0.05 and ***p < 0.001.

To characterize the immune response induced by liposomal lipid DPPC, BALB/c mice were immunized with Lp DPPC with or without encapsulated OVA. As shown in Figure 1A, Lp DPPC, in the absence of antigen, induced similar DPPC-specific IgM response in BALB/c mice to those liposomes encapsulating OVA, used as control group. There were no differences in IgM titer after one or two administrations (Figure 1B), and no IgG antibodies were detected after immunization with empty liposomes (data not shown). Thus, liposomal DPPC induced a primary antibody response that was significantly impaired in B-1 cells-deficient BALB/*xid* mice (Figure 1C), indicating the crucial role of this B cell population, as had been demonstrated for Lp DPPC-containing OVA in our previously published experiments (20) and now confirmed with both empty and antigen-encapsulating particles.

**Figure 2:**
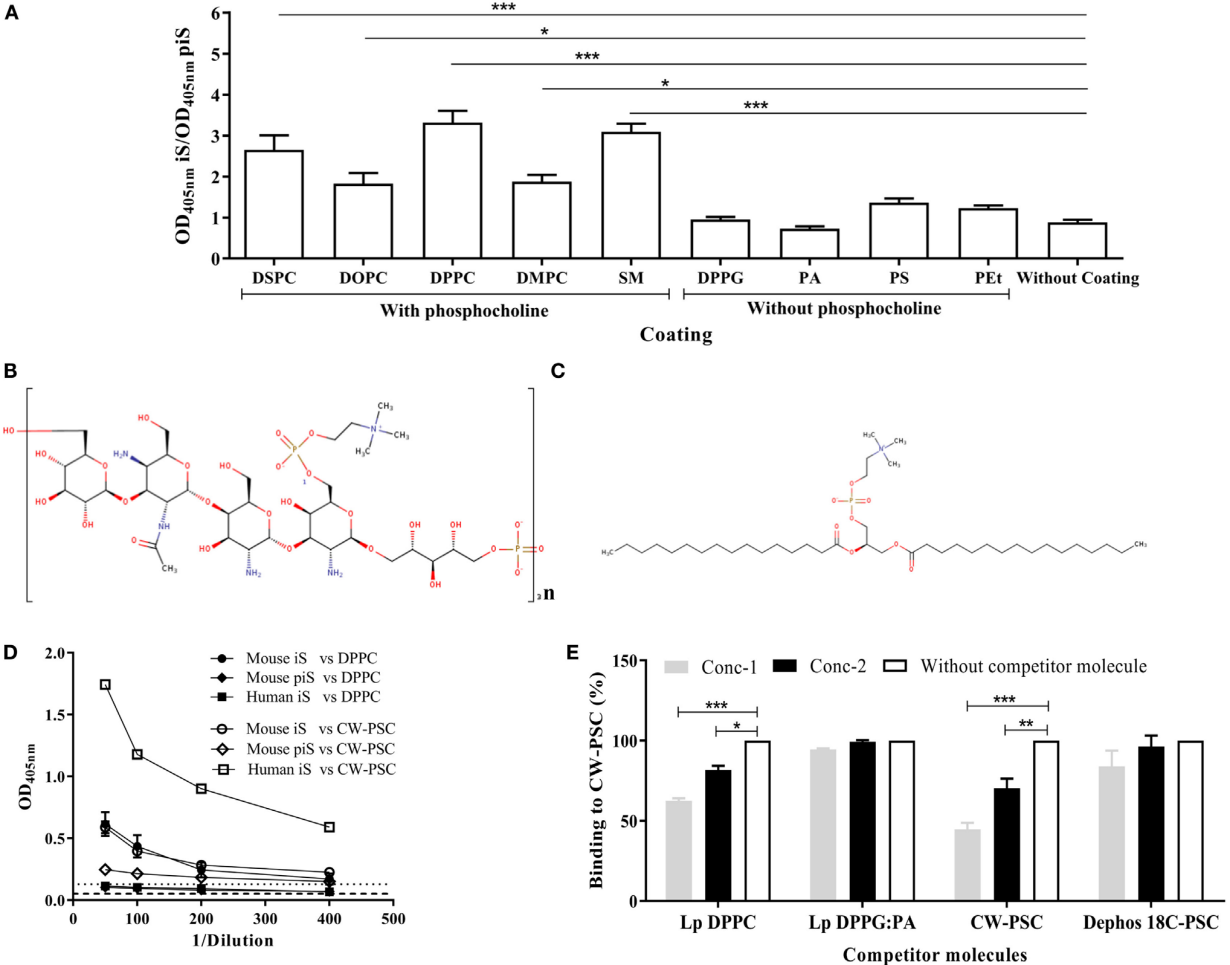
**Antibodies induced by liposomal DPPC are specific for the phosphocholine group**. **(A)** The recognition of a panel of lipids containing or not phosphocholine in their structure by sera from BALB/c mice immunized i.p with empty Lp DPPC (*n* = 11) was evaluated by ELISA. Sera were diluted 1:100, and the ratio of optical density at 405 nm between immune sera (OD_405 nm_ iS) and preimmune sera (OD_405 nm_ piS) was calculated. The following lipids were assessed: distearoyl-phosphatidylcholine (DSPC), dioleoyl-phosphatidylcholine (DOPC), dipalmitoyl-phosphatidylcholine (DPPC), dimyristoyl-phosphatidylcholine (DMPC), sphingomyelin (SM), dipalmitoyl-phosphatidylglycerol (DPPG), phosphatidic acid (PA), phosphatidylserine (PS), and phosphatidylethanolamine (PEt). The 2D structures of **(B)** the cell wall polysaccharide (CW-PSC) from *S. pneumoniae* and **(C)** DPPC were drawn using the Marvin Sketch program version 15.2.23.0 (2015), ChemAxon (http://www.chemaxon.com). **(D)** Serial dilutions of iS and piS from BALB/c mice immunized with Lp DPPC (*n* = 7) were evaluated by ELISA against DPPC and CW-PSC. A serum from a human donor immunized with a pneumococcal vaccine was used as positive control of CW-PSC recognition. Dashed and dotted lines indicate the background signal (without well coating) with mice and human serum, respectively. **(E)** The ability of Lp DPPC to inhibit the binding of DPPC-specific antibodies to CW-PSC was evaluated by a competitive ELISA. Sera from mice immunized with Lp DPPC were diluted 1:100, mixed 1:1 (v/v) with suspensions of Lp DPPC at two different concentrations (conc-1: 80 μg mL^−1^ and conc-2: 20 μg mL^−1^), and incubated for 2 h at 37°C. CW-PSC at conc-1: 50 μg mL^−1^ and conc-2: 12 μg mL^−1^ was used as positive control, while Lp DPPG:PA (a ratio 0.25:0.75) and dephos 18C PSC at the same concentrations that Lp DPPC and CW-PSC, respectively, were used as negative controls. The mixtures were added to an ELISA plate coated with CW-PSC and incubated overnight at 4°C. The percentage of binding to CW-PSC of sera in the presence of competitor molecule was calculated with respect to binding without competitor molecule. Means ± SEM are shown. Statistical analysis was performed with a Friedman test with Dunn posttest **(A)** and Kruskal–Wallis test with Dunn posttest **(E)**; *p < 0.05, **p < 0.01, and ***p < 0.001.

The reactivity of sera from mice immunized with empty Lp DPPC against a panel of lipids, containing or not phosphocholine in their structure, was evaluated. As shown in Figure 2A, the signal ratio of immune sera over preimmune sera was only significantly higher than background in the cases of lipids containing the phosphocholine group (DSPC, DOPC, DPPC, DMPC, and SM). As expected, the immune sera did not recognize the lipids without phosphocholine (DPPG, PA, PS, and PEt). In addition, to evaluate the ability of these sera to recognize phosphocholine in a non-lipid molecule, we tested reactivity with CW-PSC from *S. pneumoniae*, a polysaccharidic structure that only shares with DPPC the presence of the phosphocholine group (Figures 2B,C). As shown in Figure 2D, immune sera from mice showed similar reactivity against CW-PSC and DPPC. In contrast, a serum sample from a human donor immunized with a pneumococcal vaccine only recognized CW-PSC (Figure 2D). To confirm that the structure recognized by mouse sera was the phosphocholine group in both molecules, we carried out a competitive ELISA against CW-PSC. The previous incubation of sera with Lp DPPC inhibited binding to CW-PSC, in a concentration-dependent manner, similar to positive control CW-PSC. As expected, Lp DPPG:PA or dephos18C PSC did not affect the binding of sera to CW-PSC (Figure 2E) at any of the concentrations tested. Altogether, these results demonstrated the specificity for phosphocholine of the antibodies induced by empty Lp DPPC, which is in agreement with our previous data using OVA-encapsulating particles (20). In the present work, this finding was reinforced by using a larger panel of phosphocholine-containing molecules.

### Opsonization of Lp DPPC/OVA with Anti-Phosphocholine Antibodies-Containing Serum Total Ig Fraction Partially Mimics the B-1 Cells-Mediated Immunostimulatory Effect of This Formulation

**Figure 3:**
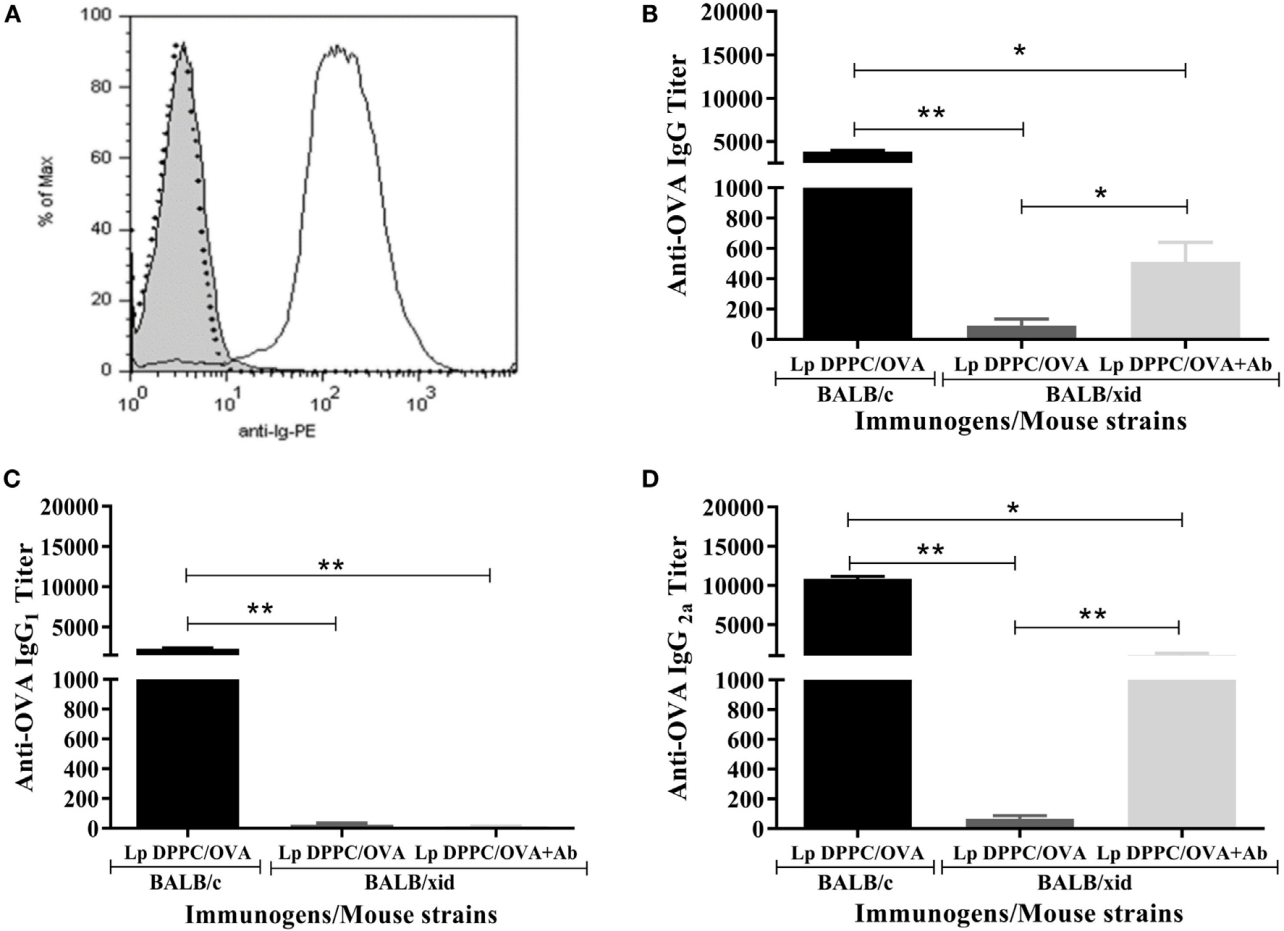
**Opsonization of Lp DPPC/OVA with anti-phosphocholine antibodies-containing serum total Ig fraction partially mimics in BALB/*xid* mice the OVA-specific antibody response induced by this liposomal formulation in wild-type animals**. **(A)** Representative histograms of Lp DPPC/OVA opsonized with anti-phosphocholine antibodies-containing serum total Ig fraction (Lp DPPC/OVA + Ab) (black line), Lp DPPC/OVA alone (filled), and Lp DPPC/OVA opsonized with an isotype-matched control antibody (dotted line). OVA-specific **(B)** IgG, **(C)** IgG1, and **(D)** IgG2a were measured by ELISA in sera from BALB/*xid* mice (*n* = 5) immunized i.p. with two doses of Lp DPPC/OVA + Ab, and BALB/*xid* (*n* = 5) and BALB/c (*n* = 6) mice immunized i.p. with two doses of Lp DPPC/OVA. Means ± SEM are shown. Statistical analysis was performed with one-way ANOVA test with Tukey posttest **(C)** or Kruskal–Wallis test with Dunn posttest **(B,D)**; *p < 0.05 and **p < 0.01.

As in our previous report (20), we confirmed that the ability of Lp DPPC to potentiate the antibody response against encapsulated OVA was impaired in BALB/*xid* mice (Figure 3). To evaluate whether anti-phosphocholine antibodies participate in the B-1 cells-mediated immunostimulatory effect of Lp DPPC, BALB/*xid* mice were immunized with Lp DPPC/OVA opsonized with the total Ig fraction from anti-DPPC antisera generated in wild-type animals (Lp DPPC/OVA + Ab). The Ig fraction ability to opsonize these particles was assessed by flow cytometry (Figure 3A). BALB/*xid* mice immunized with Lp DPPC/OVA + Ab exhibited significantly increased anti-OVA IgG titers in comparison with those induced by Lp DPPC/OVA alone, although without reaching the levels obtained in wild-type animals (Figure 3B). Whereas almost no IgG1 response was observed in any of the BALB/*xid* mouse groups (Figure 3C), the levels of IgG2a reproduced the results of the total IgG titers (Figure 3D). In summary, the opsonization with anti-phosphocholine antibodies-containing total serum Ig fraction partially rescued the immunostimulatory properties of Lp DPPC in B-1 cells-deficient mice.

### Opsonization with Phosphocholine-Specific Antibodies Increases the Uptake of Lp DPPC/OVA by Peritoneal Macrophages

**Figure 4:**
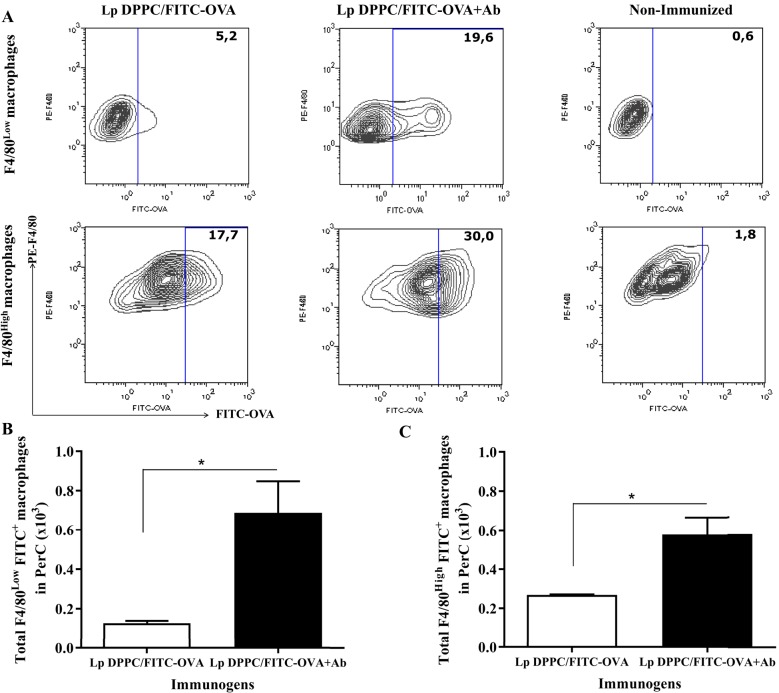
**Opsonization of Lp DPPC/OVA with anti-phosphocholine antibodies-containing serum total Ig fraction improves the *in vivo* uptake of encapsulated OVA by peritoneal macrophages**. BALB/*xid* mice (*n* = 3) were immunized i.p. with FITC-OVA encapsulated into Lp DPPC opsonized or not with anti-phosphocholine antibodies-containing serum total Ig fraction (Lp DPPC/FITC-OVA + Ab and Lp DPPC/FITC-OVA, respectively). Non-immunized BALB/*xid* mice were used as control. **(A)** Representative contour graphs of FITC-OVA vs. PE-F4/80 for both macrophage populations (F4/80^Low^ and F4/80^High^) from one representative animal per group. Means ± SEM of the total number of antigen-loaded **(B)** F4/80^Low^ and **(C)** F4/80^High^ macrophages are shown. Statistical analysis was performed with Mann–Whitney *U* test: *p < 0.05.

The more abundant large peritoneal macrophages (LPMs) express high levels of the canonical surface markers CD11b and F4/80 (F4/80^High^ macrophages), while small peritoneal macrophages (SPMs) express lower levels of these molecules (F4/80^Low^ macrophages) (26). We first assessed the uptake of Lp DPPC by both populations by immunizing BALB/*xid* mice with Lp DPPC/FITC-OVA previously opsonized or not with anti-phosphocholine antibodies-containing serum total Ig fraction. Peritoneal cells from each group were analyzed by flow cytometry following the gating strategy described in Figure S1 in Supplementary Material. BALB/*xid* mice immunized with opsonized Lp DPPC/FITC-OVA showed a higher frequency of both phenotypes of peritoneal macrophages internalizing the labeled antigen (19.6% F4/80^Low^ and 30% F4/80^High^) than in the case of animals receiving non-opsonized particles (5.2% F4/80^Low^ and 17.7% F4/80^High^) (Figure 4A). In addition, a significant increase in the total number of antigen-loaded F4/80^Low^ and F4/80^High^ peritoneal macrophages was detected in animals receiving the opsonized liposomes in comparison with those that received non-opsonized vesicles (Figures 4B,C, respectively).

**Figure 5:**
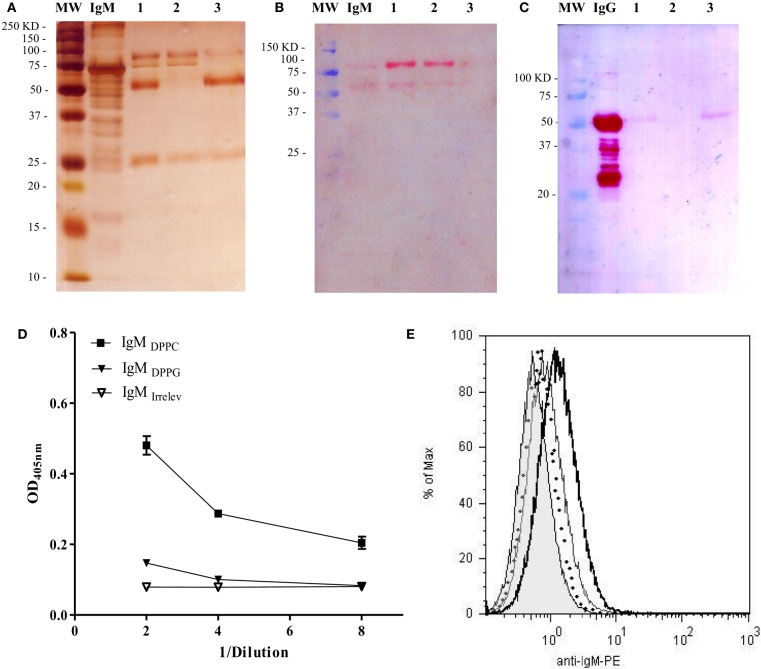
**Analysis of purity and DPPC-reactivity of IgM purified from anti-phosphocholine antibodies-containing sera**. The IgM fraction purified from sera of BALB/c mice immunized with empty Lp DPPC was analyzed by SDS-PAGE and Western blot. **(A)** Silver-stained SDS-PAGE in 12% acrylamide under reducing conditions. The presence of **(B)** IgM and **(C)** IgG in each sample was determined by Western blot using alkaline phosphatase-conjugated goat anti-mouse IgM (μ-chain-specific) and anti-mouse IgG (whole molecule) antibodies, respectively. MW: molecular weight markers; IgM/IgG: standards. 1: eluted fraction from the IgM affinity chromatography applied into the protein G chromatography; 2: unbound fraction; and 3: eluted fraction. Recognition of DPPC by IgM from sera of animals immunized with Lp DPPC (IgM_DPPC_) or Lp DPPG (IgM_DPPG_), and by an irrelevant IgM (IgM_irrelev_) was assessed by **(D)** ELISA and **(E)** flow cytometry. In **(E)**, representative histograms of Lp DPPC/OVA opsonized with IgM_DPPC_ (black line), IgM_DPPG_ (gray line), IgM_irrelev_ (dotted line), and Lp DPPC/OVA alone (filled) are shown.

In order to determine if this effect was due to IgM-specific DPPC, we purified the IgM fraction from the anti-phosphocholine antibodies-containing serum total Ig preparation (IgM_DPPC_). Two affinity chromatography steps were necessary to obtain purified IgM. The eluted fraction from the IgM affinity chromatography step still contained IgG contaminants, as shown in lines one of Figures 5A,C. When this fraction was applied into the protein G chromatographic column, a purified IgM preparation was obtained, as determined by SDS-PAGE and Western blot (Figures 5A–C, lines 2). The absence of IgG in this preparation was also verified by ELISA (data not shown). The IgM fraction from sera of animals immunized with Lp DPPG was also purified using a similar strategy (IgM_DPPG_, data not shown). Only the IgM fraction from sera of animals immunized with Lp DPPC recognized DPPC, while IgM_DPPG_ and IgM_irrelev_ were not able to bind to it (Figure 5D). The opsonization of Lp DPPC/OVA with IgM_DPPC_, IgM_DPPG_, and IgM_irrelev_ was assessed by flow cytometry (Figure 5E). As expected, the higher signal was obtained with the DPPC-specific IgM-enriched preparation.

**Figure 6:**
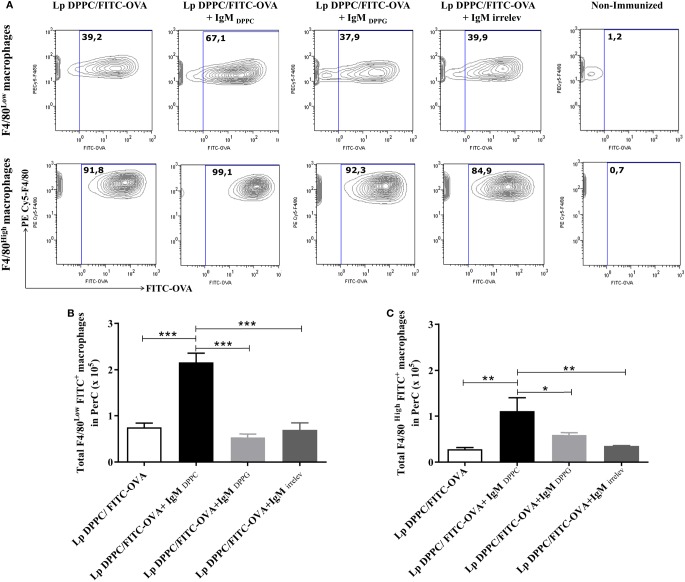
**Opsonization of Lp DPPC/OVA with anti-phosphocholine antibodies-containing serum IgM fraction improves the *in vivo* uptake of encapsulated OVA by peritoneal macrophages**. BALB/*xid* mice (*n* = 3) were immunized i.p. with FITC-OVA encapsulated into Lp DPPC (Lp DPPC/FITC-OVA) opsonized or not with the IgM fraction from sera of mice immunized with empty Lp DPPC (Lp DPPC/FITC-OVA + IgM_DPPC_) or Lp DPPG (Lp DPPC/FITC-OVA + IgM_DPPG_). Non-immunized BALB/*xid* mice and a group of mice immunized with Lp DPPC/OVA opsonized with an irrelevant IgM (Lp DPPC/FITC-OVA + IgM_irrelev_) were included as controls. **(A)** Representative contour graphs of FITC-OVA vs. PECy5-F4/80 for both macrophage populations (F4/80^Low^ and F4/80^High^) from one representative animal per group. Means ± SEM of the total number of antigen-loaded **(B)** F4/80^Low^ and **(C)** F4/80^High^ macrophages are shown. Statistical analysis was performed with one-way ANOVA test with Tukey posttest **(B)** or Kruskal–Wallis test with Dunn posttest **(C)**; *p < 0.05; **p < 0.01, and ***p < 0.001.

Finally, the uptake of opsonized Lp DPPC/FITC-OVA by peritoneal macrophages was measured in BALB/*xid* mice. In agreement with the results showed in Figure 4, the frequency of both peritoneal macrophage populations (F4/80^Low^ and F4/80^High^) loaded with the labeled antigen was higher with liposomes opsonized with IgM_DPPC_ than with Lp DPPC/FITC-OVA opsonized with IgM_DPPG_, IgM_irrelev_, or non-opsonized (Figure 6A). Moreover, the total numbers of macrophages from both populations (F4/80^Low^ and F4/80^High^) internalizing the antigen were significantly higher in mice immunized with Lp DPPC/FITC-OVA opsonized with IgM_DPPC_ than in the other groups (Figures 6B,C, respectively). Neither IgM_DPPG_ nor IgM_irrelev_ improved the uptake of liposomes by macrophages.

## Discussion

Secreted IgM is an important mediator in the optimal initiation of primary thymus-dependent humoral immune responses. It serves as a natural adjuvant by enhancing the immunogenicity of protein antigens, perhaps as a result of its ability to facilitate antigen deposition onto follicular dendritic cells and to promote rapid germinal center formation (1, 4, 27–29). Besides, the complex antigen–IgM is involved in affinity maturation (3, 28). It has also been reported that secreted IgM influences BCR signaling and promotes survival of splenic B cells (30).

We have previously shown that B-1 cells contribute to the ability of DPPC-containing liposomes to enhance the encapsulated antigen-specific antibody response. Besides, liposomal DPPC stimulates B-1 cells to produce IgM, specific for the phosphocholine polar head (20). We therefore addressed here whether these antibodies contributed to the immunostimulatory properties of Lp DPPC. The results described in the present work demonstrate that these particles were able to induce similar levels of DPPC-specific antibodies irrespective of the presence of encapsulated OVA. This response depended on B-1 cells, since it was significantly reduced in B-1 cells-deficient BALB/*xid* mice, as we previously reported for liposomes encapsulating OVA (20). *Xid* mice have extensively been used as a model of B-1 cell deficiency (13), and although these animals exhibit defects also in the B-2 cell compartment, higher doses of soluble OVA than the one we used in our work (2 μg) induced similar IgG titers in BALB/c and BALB/*xid* mice (our unpublished data), suggesting that the B-2 cell response against this antigen is not affected in the latter animals. Moreover, no significant differences have been found in the marginal zone B cell population between these two mouse strains (31).

The recognition of different lipid species containing phosphocholine in their structure, as well as the CW-PSC from *S. pneumoniae* by the antibodies induced by liposomal DPPC corroborated their specificity for the phosphocholine group. On the other hand, the lack of reactivity with DPPC of the serum from a human donor immunized with a pneumococcal vaccine is in agreement with previous results in which rabbits immunized with this antigen conjugated to bovine serum albumin elicited antibodies that recognized the saccharide moiety but not the phosphocholine group (32).

The opsonization of Lp DPPC/OVA with phosphocholine-specific antibodies partially mimicked the immunostimulatory effect of DPPC-liposomes in the OVA-specific humoral response, as proven in B-1 cells-deficient mice. Our results extend previous observations demonstrating a role for B-1 cells-derived IgM in the enhancement of IgG production by B-2 cells (28, 33).

IgM antibodies can promote humoral immune responses through complement activation (34) and engagement of receptors, such as mannan-binding lectin (35), the polymeric Ig receptor (36), the Fc alpha/mu receptor (Fcα/μR) (37, 38), or the Fc receptor specific for IgM (FcμR) (38–41). The potential roles of different cellular receptors for IgM are a topic of active investigation. The Fcα/μR is constitutively expressed on macrophages, in addition of other cells, such as B cells and follicular dendritic cells, and recognizes IgM and IgA with high and intermediate affinity, respectively (37, 42). It mediates endocytosis of IgM-coated microbes (37, 43). On the other hand, the FcμR is expressed on macrophages and dendritic cells, although to a lesser extent in comparison with other immune cells like T and B lymphocytes (29, 39–42, 44). It plays an essential role in humoral immune responses to both thymus-dependent and -independent antigens (38, 44) and acts as an endocytic receptor, internalizing antigen–IgM complex (45). The engagement of this pathway could result in synergistic activation of B cells stimulated through the BCR (46).

Liposomes coated with the anti-phosphocholine IgM produced by B-1 cells could be taken up more efficiently by antigen-presenting cells through IgM-specific receptors, thus enhancing the presentation of encapsulated antigens. Particularly in this work, we demonstrated that administration of liposomes opsonized with anti-phosphocholine antibodies-containing serum total Ig or IgM fractions enhanced the uptake of the antigen by both the large and the small populations of peritoneal macrophages of BALB/*xid* mice, whose phagocytic activity *in vivo* has been demonstrated (26). This result is in agreement with other works showing that the incubation with polyclonal IgM enhances the phagocytosis of apoptotic cells in the lungs by alveolar macrophages (47) and promotes the clearance of apoptotic microparticles released from dying cells (48). The increase in the opsonized Lp DPPC/OVA uptake by macrophages *in vivo* could be related to the improvement of the OVA-specific antibody response observed in BALB/*xid* mice immunized with this preparation. Interestingly, this humoral response was not completely restored in comparison with wild-type animals. Notably, B-1 cells-deficient animals were persistently unable to produce specific IgG1, despite the restoration of the IgG2a levels. This could be due to the role of these cells as IL-10 producers, which inhibits IgG2a and favors IgG1 production (49). This observation suggests the direct participation of B-1 cells in the adjuvanticity of liposomes beyond the production of anti-DPPC IgM. In agreement with this, we previously described the ability of B-1 cells to uptake and transport the antigen from the PerC to the spleen after intraperitoneal immunization with Lp DPPC/OVA (20).

In conclusion, in the present work, it has been demonstrated for the first time that B-1 cells-derived phosphocholine-specific antibodies induced by liposomal DPPC contribute to the immunostimulatory properties of these particles.

## Author Contributions

YC-L: conception and design of the work; acquisition, analysis, and interpretation of the data and writing of the manuscript. AL-R: analysis and interpretation of the data and critical revision of the manuscript. IL-G and YH: acquisition, analysis, and interpretation of data. CA: analysis and interpretation of the data and revision of the manuscript. RP: conception of the work, critical scientific support, interpretation of the data, revision of the manuscript, and final approval of the manuscript. MEL: conception and design of the work and critical revision and final approval of the manuscript.

## Conflict of Interest Statement

The authors declare that the research was conducted in the absence of any commercial or financial relationships that could be construed as a potential conflict of interest.

## Abbreviations

APC, allophycocyanin; Chol, cholesterol; CW-PSC, cell wall polysaccharide; DMPC, dimyristoyl-phosphatidylcholine; DOPC, dioleoyl-phosphatidylcholine; DPPC, dipalmitoyl-phosphatidylcholine; DPPG, dipalmitoyl-phosphatidylglycerol; DRV, dehydration and rehydration vesicles; DSPC, distearoyl-phosphatidylcholine; ELISA, enzyme-linked immunosorbent assay; FITC, fluorescein isothiocyanate; i.p., intraperitoneal; Lp DPPC, DPPC and chol-containing liposomes; Lp DPPG, DPPG and chol-containing liposomes; Lp DPPC/OVA, DPPC and chol-containing liposomes encapsulating OVA; LPM, large peritoneal macrophages; OVA, ovalbumin; OVA-FITC, ovalbumin labeled with fluorescein isothiocyanate; PA, phosphatidic acid; PB, phosphate buffer; PerC, peritoneal cavity; PE Cy5.5, cyanine dye (Cy5.5) combined with phycoerythrin; PE, phycoerythrin; PEt, phosphatidylethanolamine; PS, phosphatidylserine; SM, sphingomyelin; SPM, small peritoneal macrophages; SUV, small unilamellar vesicles; Xid, X-linked immunodeficient.
