# Supplementary material for: Phosphocholine-Specific Antibodies Improve T-Dependent Antibody Responses against OVA Encapsulated into Phosphatidylcholine-Containing Liposomes
Source: Front Immunol. 2016 Sep 22;7:374. doi: 10.3389/fimmu.2016.00374 (PMC5031597; doi:10.3389/fimmu.2016.00374)
Supplement: Supplementary file 1 [file image_1.pdf]

Supplementary Material

Phosphocholine-specific antibodies improve T-dependent antibody responses against OVA encapsulated into phosphatidylcholine-containing liposomes

Authors: Yoelys Cruz-Leal[1], Alejandro López-Requena[2 ^], Isbel Lopetegui-González[3], Yoan Machado[4 §], Carlos Alvarez[1], Rolando Pérez[2*] and María E. Lanio[1*]

Corresponding author: Dr. María Eliana Lanio, mlanio@fbio.uh.cu and Dr. Rolando Pérez rolando@cim.sld.cu

Supplementary Figure

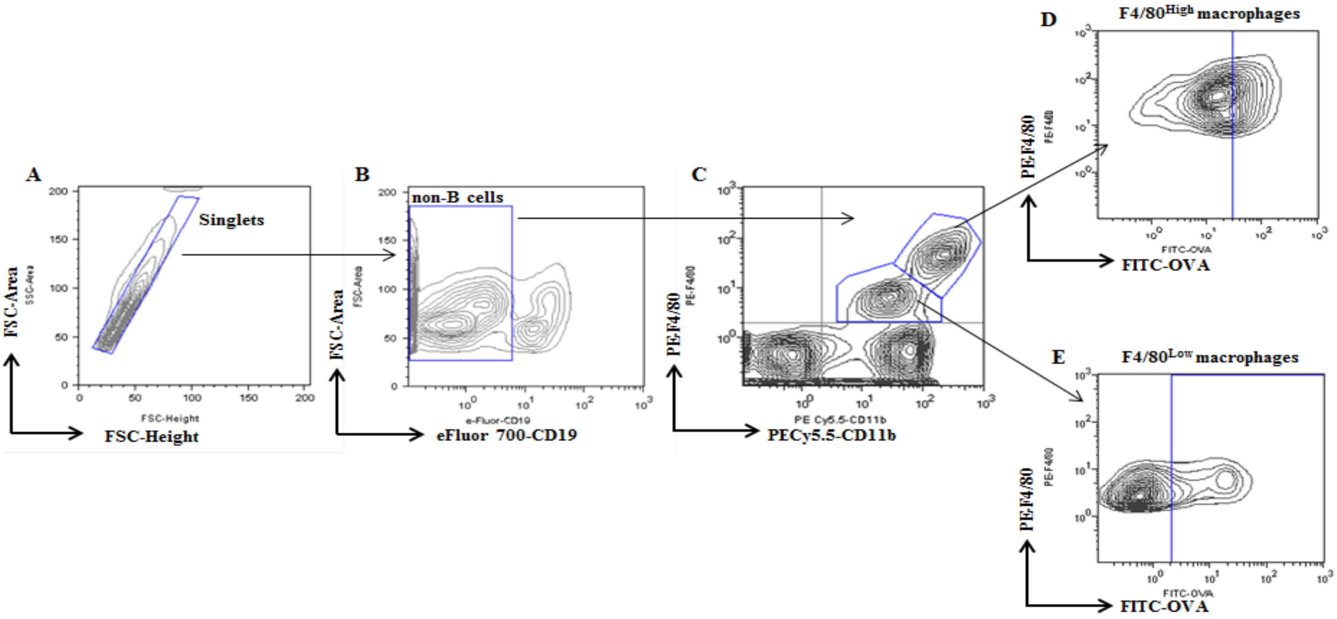

Supplementary Figure 1. Gating strategy used to identify peritoneal macrophages internalizing FITC-OVA encapsulated into DPPC-liposomes. Cells from peritoneal wash of BALB/c mice immunized with Lp DPPC/FITC-OVA were stained with different fluorochrome-labeled antibodies: PE-F4/80; PE Cy5.5-CD11b and eFluor700-CD19 and analyzed by flow cytometry. (**A**) Forward scatter-height (FSC-Height) vs. forward scatter-area (FSC-Area) contour graph to exclude doublets. (**B**) eFluor700-CD19 vs. FSC-Area contour graph to exclude B cells. (**C**) PE Cy5.5-CD11b vs. PE-F4/80 contour graph from non-B cells to identify peritoneal macrophage

populations: F4/80^{High} and F4/80^{Low} macrophages. (**D** and **E**) FITC-OVA vs. PE-F4/80 contour graphs from F4/80^{High} and F4/80^{Low} macrophages to identify F4/80^{High}FITC^{+} and F4/80^{Low}FITC^{+} cells, respectively. Data are representative from at least two experiments.
